# *EGFR* Mutation is a Prognostic Factor in Lung Cancer Patients with Pleural Dissemination Detected During or After Surgery

**DOI:** 10.1245/s10434-023-13791-y

**Published:** 2023-06-25

**Authors:** Toshiya Fujiwara, Kazuhiko Shien, Motoki Matsuura, Junichi Soh, Hiromasa Yamamoto, Soshi Takao, Yuho Maki, Tsuyoshi Ueno, Ryujiro Sugimoto, Ken Suzawa, Mikio Okazaki, Hiroyuki Tao, Makio Hayama, Masafumi Kataoka, Yoshifumi Sano, Hidetoshi Inokawa, Motohiro Yamashita, Osamu Kawamata, Kazuhiko Kataoka, Shinichi Toyooka

**Affiliations:** 1 Department of Thoracic Surgery, Hiroshima City Hiroshima Citizens Hospital, Hiroshima, Japan; 2 Okayama University Thoracic Surgery Study Group (OUTSSG), Okayama, Japan; 3 Department of Thoracic Surgery, Okayama University Hospital, Okayama, Japan; 4 Department of Epidemiology, Graduate School of Medicine, Dentistry and Pharmaceutical Sciences, Okayama University, Okayama, Japan; 5 Department of Thoracic Surgery, Shikoku Cancer Center, Matsuyama, Japan; 6 Center of Chest Medicine and Surgery, Ehime University, Toon, Japan; 7 Department of Thoracic Surgery, Japanese Red Cross Society Himeji Hospital, Himeji, Japan; 8 Department of Thoracic Surgery, Japanese Red Cross Okayama Hospital, Okayama, Japan; 9 Department of Surgery, Okayama Saiseikai General Hospital, Okayama, Japan; 10 Division of Thoracic Surgery, Yamaguchi-Ube Medical Center, Ube, Japan; 11 Department of Surgery, Onomichi Municipal Hospital, Onomichi, Japan; 12 Department of Thoracic Surgery, Iwakuni Clinical Center, Iwakuni, Japan

## Abstract

**Background:**

Primary lung tumors are sometimes resected when either pleural dissemination (PD) or malignant pleural effusion (MPE) exists. This study clarified the prognostic factors for non-small cell lung cancer (NSCLC) with either PD and MPE, or both, detected during or after surgery.

**Patients and Methods:**

We examined patients with NSCLC from a multicenter database who had either PD, MPE, or both, detected during or after surgery between 2005 and 2015. Hazard ratios and 95% confidence intervals were estimated using the Cox proportional hazards model adjusted for potential confounding factors.

**Results:**

Among 9463 registered patients, PD, MPE, or both, were found in 114 patients with NSCLC during or after surgery. Primary tumor resection and exploratory thoracotomy were performed in 65 and 49 patients, respectively. In univariate analysis, adenocarcinoma, clinically undetected lymph node metastasis (c-N0 or unknown), *EGFR* mutation, and combination of chemotherapy or tyrosine kinase inhibitors after surgery were better prognostic factors for overall survival (OS), whereas in the multivariate analysis, adenocarcinoma, clinically undetected lymph node metastasis, and *EGFR* mutation were favorable independent prognostic factors in OS. Additionally, limited to patients with *EGFR* mutation, patients with primary lung tumor resection showed a significantly better 5-year OS than those with exploratory thoracotomy (86.4 vs. 44.8%; *p* < 0.001).

**Conclusion:**

Our findings show that surgical resection of primary tumors could improve the prognosis of patients with PD, MPE, or both, detected during or after surgery when the tumors harbor an *EGFR* mutation.

**Supplementary Information:**

The online version contains supplementary material available at 10.1245/s10434-023-13791-y.

Non-small cell lung cancer (NSCLC) with pleural dissemination (PD) or malignant pleural effusion (MPE) is classified as stage IVA in the 8th edition of TNM staging, and radical resection is not indicated. Among patients who should be operable before surgery, 1–3% of cases are accompanied with PD or MPE.^1,2^ When PD or MPE is found during surgery, the primary tumor is sometimes resected for histological examination, genetic testing, and symptom relief, although its prognostic impact is unclear. However, among these PDs or MPEs, or both, long-term survivors exist. The presence of driver mutation in the *EGFR* in patients with NSCLC are detected in 30–50% of East Asians. The efficacy of several clinical trials on EGFR-tyrosine kinase inhibitors (TKIs) has recently been demonstrated. Additionally, EGFR-TKI is now in its third-generation and treatment options are expanding.

This study clarifies the prognostic factors for stage IVA patients with NSCLC diagnosed with PE or MPE during or after surgery, and the impact of surgical resection on prognosis through a retrospective review of a multicenter database.

## Materials and Methods

### Patients and Methods

This study protocol was approved by the Ethics Committee of Okayama University Hospital (No. 1804-040, 24 April 2018), and every joint research facility also obtained permission from each Ethics Commission. Written informed consent from each patient was waived, and all methods in this study were in compliance with the relevant guidelines and regulations. We extracted 114 Japanese patients (1.2%) of stage IVA patients with NSCLC whose PD or MPE was found during or after surgery, from 9463 patients with NSCLC registered in the Okayama University Thoracic Surgery Study Group (OUTSSG) multicenter database of nine hospitals belonging to OUTSSG, from January 2005 to December 2015. The patients’ clinicopathological factors are shown in Table 1. The medical records were reviewed and analyzed retrospectively, and the degree of PD was classified as follows: D0, MPE without disseminated nodules; D1, 1–5 disseminated nodules; D2, > 5 disseminated nodules. Since thoracic lavage cytology-positive cases were not reflected in the clinical stage, they were excluded. OS was defined as the day of surgery to the day of death or the last follow-up day.

**Table 1:** Patient characteristics

Variables	*n* = 114
Median age, years (range)	70 (39–89)
Sex, male/female	68/46
Smoking history, current/former/never	22/45/47
Histology, ad/sq/others	95/9/10
Tumor location, RUL/RML/RLL/LUL/LLL	24/11/29/23/27
cN, N0/N-positive/unknown	70/43/1
c-Stage, IA/IB/IIA/IIB/IIIA/IIIB/IV	21/28/16/10/17/2/20
Procedure, primary tumor resection/exploratory thoracotomy	65/49
Operation, lobectomy or bi-lobectomy/segmentectomy/wedge resection	41/8/16
Additional procedure during operation,^a^ yes/no	20/94
D factor, D0/D1/D2	12/41/61
PD and/or MPE detection, intraoperative/permanent histology	101/13
*EGFR*, wild-type/Del 19/L858R/unknown	42/25/16/31
Treatment after surgery, chemotherapy or TKI/BSC/unknown	90/16/8

*Ad* adenocarcinoma, *Sq* squamous cell carcinoma, *RUL* right upper lobe, *RML* right middle lobe, *RLL* right lower lobe, *LUL* left upper lobe, *LLL* left lower lobe, *D0* malignant pleural effusion without disseminated nodules, *D1* 1–5 disseminated nodules, *D2* > 5 disseminated nodules, *PD* pleural dissemination, *MPE* malignant pleural effusion, *Del* deletion, *TKI* tyrosine kinase inhibitor, *BSC* best supportive care

^a^Addition of distilled water perfusion therapy with/without a chemotherapeutic agent intraoperatively

### Statistical Analyses

For comparing the two groups, continuous variables were analyzed using Student’s *t*-test, and categorical variables were analyzed using Pearson’s Chi-square test. Kaplan–Meier survival analysis examined clinicopathological factors that influence OS, and prognostic factors affecting survival were evaluated using the log-rank test for univariable analysis. The Cox proportional hazard model was used to conduct a multivariate analysis adjusted for variables that showed *p <* 0.05 in univariable measurement and the previously reported prognostic factors in NSCLC. Hazard ratios (HRs) and 95% confidence intervals (CIs) were estimated. All significant difference tests were performed using a two-tailed test, and *p*-values < 0.05 were considered statistically significant. JMP software version 16 (SAS Institute Inc., Cary, NC, USA) was used to perform all statistical analyses, and GraphPad Prism version 6.0 (GraphPad Software Inc., La Jolla, CA, USA) was used for graphic display.

## Results

### Patient Characteristics

The characteristics of the patients are shown in Table 1. In 65 patients (57.0%), primary lung tumor resection was performed, whereas 49 patients (43.0%) had only exploratory thoracotomy. PD, MPE, or both, were detected intraoperatively in 101 patients (88.6%). In 13 patients (11.4%), PD that was not detected macroscopically during surgery was diagnosed pathologically in the permanent specimen postoperatively. As an intraoperative additional therapy, distilled water perfusion therapy (chemotherapeutic agent combined/non-combined) was administered to 20 patients (17.5%). *EGFR* mutation was investigated in 83 patients; *EGFR* mutation was found in 41 patients (exon 19 deletions in 25 patients and L858R in 16 patients) and 42 were detected to be wild-type.

### Survival Analyses

**Fig. 1:**
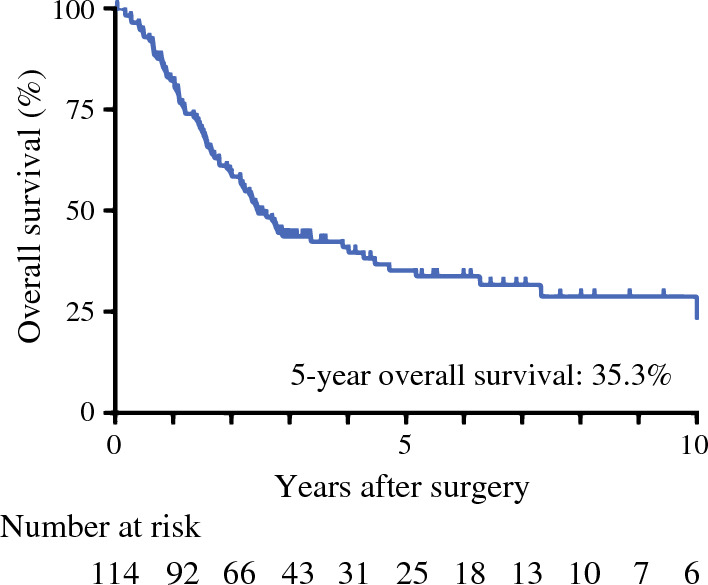
Overall survival

The median observation period was 28.6 months and the 5-year OS was 35.3% (Fig. 1). The 5-year OS was not significantly different between the primary lung tumor-resected group and the exploratory thoracotomy group (40.9 vs. 28.1%; *p* = 0.247) [Table 2]. Adenocarcinoma, clinically undetected lymph node metastasis (cN0 or unknown), *EGFR* mutation, and the combination of postoperative chemotherapy or TKI were identified as better prognostic factors in OS in univariate analysis (Table 2). In the multivariate analysis, adenocarcinoma (HR 0.356, 95% CI 0.188–0.674), clinically undetected lymph node metastasis (HR 0.427, 95% CI 0.263–0.692), and *EGFR* mutation (HR 0.263, 95% CI 0.139–0.500) were favorable independent prognostic factors (Table 2).

**Table 2:** Univariate and multivariate analysis of prognostic factors

Variables	Univariate			Multivariate				
	*n*	5-year OS (%)	*p*-Value	HR	95% CI			*p*-Value
Sex			0.068					0.900
Male	68	25.3		0.966	0.565 to 1.652			
Female	46	49.6		1.000				
Histology			< 0.001					0.002
Ad	95	41.8		0.356	0.188 to 0.674			
Non-ad	19	0		1.000				
cN			< 0.001					< 0.001
N0 or unknown	71	48.2		0.427	0.263 to 0.692			
N-positive	43	13.0		1.000				
Clinical stage			0.151	–				–
IA	21	42.3						
IB	28	48.0						
IIA	16	8.1						
IIB	10	30.0						
IIIA	17	41.2						
IIIB	2	30.0						
IV	20	25.0						
Procedure			0.247					0.148
Exploratory thoracotomy	49	28.1		1.000				
Primary tumor resection	65	40.9		0.663	0.380 to 1.157			
Additional procedure during operation^a^			0.0651					0.593
No	94	31.9		1.000				
Yes	20	48.9		0.804	0.361 to 1.789			
D factor			0.271					0.587
D0	12	27.5		1.000				
D1/D2	102	35.9		0.785	0.329 to 1.876			
PD and/or MPE detection			0.795	–				–
Intraoperative	101	36.3						
Permanent histology	13	28.9						
*EGFR* mutation			< 0.001					< 0.001
Wild-type or unknown	73	18.1		1.000				
Mutant	41	64.9		0.263	0.139 to 0. 500			
Treatment after surgery			0.001					0.079
BSC or unknown	24	19.3		1.000				
Chemotherapy or TKI	90	39.0		0.586	0.323 to 1.063			

*Ad* adenocarcinoma, *D0* malignant pleural effusion without disseminated nodules, *D1* 1–5 disseminated nodules, *D2* > 5 disseminated nodules, *PD* pleural dissemination, *MPE* malignant pleural effusion, *BSC* best supportive care, *TKI* tyrosine kinase inhibitor

^a^Addition of distilled water perfusion therapy with/without a chemotherapeutic agent intraoperatively

### Subgroup Analyses of Patients with *EGFR* Mutation

**Fig. 2:**
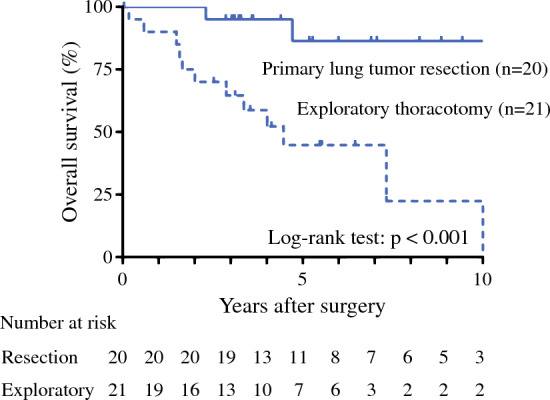
Survival analysis in *EGFR*-mutant patients stratified by surgical procedure

We performed a survival analysis by limiting the analysis to 41 patients with *EGFR* mutation. The clinicopathological characteristics of patients with *EGFR* mutation in the primary lung tumor-resected group (*n* = 20) and exploratory thoracotomy group (*n* = 21) are shown in electronic supplementary Table 1. When comparing the exploratory thoracotomy group with the primary lung tumor-resected group, the number of patients who received additional distilled water perfusion therapy intraoperatively was significantly higher in the primary tumor resection group, and the number of D2-positive patients was significantly higher in the exploratory thoracotomy group. EGFR-TKI was administered to 18 patients in the resected group and 15 patients in the exploratory thoracotomy group. The median duration of observation was 48.1 months. The 5-year OS of the resected group was 86.4%, compared with 44.8% in the exploratory thoracotomy group, and patients with primary lung tumor resection showed a significantly better 5-year OS than those with exploratory thoracotomy (*p* < 0.001) [Fig. 2]. On the other hand, the 5-year OS of patients with *EGFR* wild-type was 27.8% in the resected group (*n* = 24) and 25.1% in the exploratory thoracotomy group (*n* = 18) [*p* = 0.773].

## Discussion

Although surgical resection of stage IVA NSCLC with PD, MPE, or both, is not generally recommended, we have seen cases of long-term survival with treatment. Some of these cases involved patients who underwent primary lung tumor resection and chemotherapy; however, it is unclear which factors influence long-term survival. Several studies have found that carcinomatous pleuritis found during surgery has a relatively good prognosis.^1–13^ According to the lung cancer registry for resected cases, by the Japan Lung Cancer Society,^1^ carcinomatous pleuritis was found in 2.9% of cases. The median survival was 34 months, with a 5-year OS of 29.3%.

Platinum-based chemotherapy is the standard treatment for PD or MPE.^14^ Chemotherapy was used to treat approximately 80% of patients in this study. Similarly, molecularly targeted therapeutics have recently been frequently chosen based on gene alteration^15–18^. Ichinose et al.^19^ reported that an intrathoracic distilled water-cisplatin perfusion effectively controlled pleural effusion. Twenty patients (17.5%) were included in this study, and while there was no significant difference, the prognosis tended to be favorable.

In this study, *EGFR* mutation with resection of the primary tumor was an independent factor of better prognosis in multivariate analysis. However, as a novel finding, surgical resection of the primary tumor showed a significantly favorable prognosis of patients with *EGFR*-mutant NSCLC compared with exploratory thoracotomy. This phenomenon was not observed in patients with wild-type *EGFR*. While the reason for these results was unclear, tumor volume reduction by resection of the primary site and EGFR-TKI treatment could synergize patient prognosis with *EGFR*-mutant NSCLC. Regarding NSCLC with *ALK* rearrangement, which is considered a driver mutation like *EGFR* mutation, the number of patients was small and conclusive results were difficult to obtain (data not shown).

A limitation of this study is that it was conducted retrospectively. Undertaking prospective studies is challenging because it is difficult to register cases where PD is not suspected before surgery. Second, because this was a collaborative multicenter study, there was some measurement misclassification in the surgical procedure depending on the facility. Therefore, pathological and clinical staging are mixed, and staging migration may exist. However, these non-differential misclassifications could distort our results only toward the null. Thus, our estimated HR for EGFR mutation might not be fully explained by these biases and might partly reflect the true association. Third, the case registry period was 11 years, which was excessively long. Finally, as chemotherapy progresses, the postoperative treatment type has evolved. Given that treatment targeting gene mutations had a significant impact on prognosis, we primarily chose the era in which EGFR-TKI was widely used in clinical practice.

## Conclusion

Our findings show that surgical resection of primary tumors could improve the prognosis of patients with PD, MPE, or both, detected during or after surgery when the tumors harbor an *EGFR* mutation. Large-scale studies over a short period of time and in more facilities should confirm this.

## Supplementary Information

Below is the link to the electronic supplementary material.

Supplementary file1 (DOCX 26 KB)
