# Supplementary material for: EGFR Mutation is a Prognostic Factor in Lung Cancer Patients with Pleural Dissemination Detected During or After Surgery
Source: Ann Surg Oncol. 2023 Jun 25;30(11):6697–702. doi: 10.1245/s10434-023-13791-y (PMC10506925; doi:10.1245/s10434-023-13791-y)
Supplement: Supplementary file 1 — Supplementary file1 (DOCX 26 KB) [file 10434_2023_13791_MOESM1_ESM.docx]

**Supplementary Table 1. Patient characteristics of *EGFR* mutant patients**

| **Variables** | **Primary tumor resection**  **(n=20)** | **Exploratory thoracotomy**  **(n=21)** | ***P* value** |
| --- | --- | --- | --- |
| Median age (year) | 65 | 65 | 0.821 |
| Sex, Male / Female | 6 / 14 | 11 / 10 | 0.146 |
| Smoking history, Current / Former / Never | 2 / 4 / 14 | 2 / 7 / 12 | 0.622 |
| Histology, Ad / non-Ad | 20 / 0 | 21 / 0 | - |
| Tumor location, RUL / RML / RLL / LUL / LLL | 5 / 2 / 4 / 5 / 4 | 5 / 3 / 6 / 5 / 2 | 0.871 |
| c-N, N0 / N-positive | 15 / 5 | 13 / 8 | 0.368 |
| c-Stage, IA / IB / IIA / IIB / IIIA / IIIB / IV | 5 / 7 / 1 / 2 / 4 / 1 / 0 | 3 / 4 / 1 / 2 / 2 / 0 / 9 | 0.063 |
| Additional procedure during operation*,  Yes / No | 9 / 11 | 0 / 21 | <0.001 |
| D factor, D0 / D1 / D2 | 3 / 8 / 9 | 0 / 1 / 20 | 0.002 |
| PD and/or MPE detection,  Intraoperative / Permanent histology | 17 / 3 | 21 / 0 | 0.065 |
| Treatment after surgery,  Chemotherapy or TKI / BSC or Unknown | 18 / 2 | 18 / 3 | 0.675 |
| Administration of EGFR-TKI,  Yes / No or Unknown | 18 / 2 | 15 / 6 | 0.134 |

Ad; adenocarcinoma, RUL; right upper lobe, RML; right middle lobe, RLL; right lower lobe, LUL; left upper lobe, LLL; left lower lobe, D0; malignant pleural effusion without disseminated nodules, D1; 1-5 disseminated nodules, D2; >5 disseminated nodules, BSC, best supportive care, TKI; tyrosine kinase inhibitor

*Addition of distilled water perfusion therapy with/without chemotherapeutic agent intra-operatively
